# Spontaneous Pneumomediastinum Induced by a Combination of Flu-A Infection and E-cigarettes: A Case Report

**DOI:** 10.7759/cureus.61689

**Published:** 2024-06-04

**Authors:** Jason C Sanchez, Jaron Sanchez, Farah R Farah

**Affiliations:** 1 Hospital Medicine, University of the Incarnate Word School of Osteopathic Medicine, San Antonio, USA; 2 Hospital Medicine, Nova Southeastern University Dr. Kiran C. Patel College of Osteopathic Medicine, Clearwater, USA; 3 Hospital Medicine, Methodist Health System, San Antonio, USA

**Keywords:** pulmonary barotrauma, electronic cigarettes (e-cigarettes), subcutaneous emphysema, spontaneous pneumomediastinum (spm), pneumomediastinum (pm)

## Abstract

Pneumomediastinum (PM) and subcutaneous emphysema are characterized by extra-alveolar air within the mediastinum and subcutaneous tissue. PM may occur spontaneously or due to trauma or an underlying airway disease. Spontaneous pneumomediastinum (SPM) may be caused by intractable vomiting, forceful coughing, child birthing, or performing a Valsalva maneuver. However, there are limited studies or case reports that present a combination of influenza A infection and electronic cigarette (e-cigarette)-induced SPM. This case report presents SPM in a previously healthy 20-year-old female with untreated influenza A infection and a history of e-cigarette use who presented to the emergency department with fever, cough, chest pain, dyspnea, and vomiting. Her physical examination was significant for neck tenderness, subcutaneous neck crepitus, and increased respiratory effort. Diagnostic evaluation included a chest X-ray and chest computed tomography that revealed PM with subcutaneous emphysema extending into the neck, as well as a negative Gastrografin study. She was treated conservatively and discharged after two days, with a follow-up scheduled at a pulmonary clinic. This case report highlights the need for a detailed substance use history, particularly e-cigarette use, when determining the etiology of SPM in a previously healthy patient. Management for SPM is conservative and should include addressing underlying etiologies with special attention to cessation and education of e-cigarettes and illicit substances.

## Introduction

Pneumomediastinum (PM) and subcutaneous emphysema are characterized by extra-alveolar air within the mediastinum and subcutaneous tissue [1]. PM may occur spontaneously or due to trauma or an underlying airway disease. Common causes for the latter include blunt or direct penetrating trauma, iatrogenic injury, Boerhaave’s syndrome, perforated duodenal ulcer, ulcerative colitis, or sigmoid diverticulitis, or from an underlying airway disease [1,2]. In spontaneous pneumomediastinum (SPM), increased intra-alveolar pressure or reduced interstitial pressure causes alveoli to rupture, which introduces air into the perivascular sheaths, escaping into the mediastinum, and it may decompress into the neck, subcutaneous tissue, or abdomen [1,2].

Patients with SPM may present with chest pain, dyspnea, neck pain or swelling, dysphagia, and subcutaneous crepitations. Physical examination may demonstrate neck crepitations on palpation or auscultation, facial and cervical edema, and a “mediastinal crunch” on cardiac auscultation, known as Hamman’s sign [3]. Diagnostic evaluation includes a chest radiograph demonstrating lucent streaks, computed tomography (CT) of air outlining mediastinal structures, and an esophagram to rule out esophageal perforation [4]. SPM is a benign, self-limited condition that resolves between two to fifteen days with conservative management and management of underlying causes. It has an excellent prognosis with a very low risk of reoccurrence [5].

Historically, SPM may be caused by intractable vomiting, forceful coughing, child birthing, or performing a Valsalva maneuver [1,2]. More recently, there have been reports of e-cigarette-induced or vaping-induced SPM [6-9]. Vaping, as a form of e-cigarette, has increased in use, with 4.5% of current users being adults aged 18 years old and over and 11% of young adults aged 18-24 years old among the highest users [10]. Here, we present a case of a young, previously healthy 20-year-old female with untreated influenza A infection and a known history of e-cigarette use diagnosed with SPM.

## Case presentation

We report a 20-year-old previously healthy female with a history of pneumonia in 2021. She presented to the emergency department (ED) after two days of gradual onset of shortness of breath, cough, wheezing, and sore throat associated with fever, chills, fatigue, body aches, pressure-like chest pain, nausea, and multiple episodes of non-bloody, nonbilious emesis. The day before, she was seen at another ED and diagnosed with a viral illness, to which she was prescribed steroids with no relief in her symptoms. She has no history of recent trauma, surgeries, strenuous activity or exercise, history of breathing difficulties, inhaler use, or diagnoses of cardiopulmonary diseases. Her family history was not significant. She does not smoke tobacco but uses e-cigarettes. She also consumes alcohol and reports marijuana use with a bong apparatus two weeks before. There was no documentation of the habits surrounding e-cigarettes and marijuana use, as well as whether there was concurrent tetrahydrocannabinol (THC) use or if other illicit substances were involved.

In the ED, the patient was presented with a temperature of 100.5 °F, blood pressure of 112/60 mmHg, heart rate of 123 beats per minute, respiratory rate of 18 breaths per minute, and oxygen saturation of 94% on room air. On physical examination, she was in moderate distress with mild dehydration and had mild pharyngeal erythema, neck tenderness without masses or swelling, subcutaneous neck crepitus, increased respiratory effort with clear lungs, and tachycardia. Labs were reassuring, with a complete blood count within normal limits and no significant electrolyte derangements. She was negative for a monoscreen, SARS-CoV-2 RNA nucleic acid amplification test, and Group A Strep antigen on a rapid strep test with no growth on cultures. However, she tested positive for influenza A antigen on a rapid influenza immunoassay. Blood cultures were negative. Urinalysis was negative for nitrites and leukocyte esterases. The electrocardiogram showed sinus tachycardia with a rate of 115.

**Figure 1:**
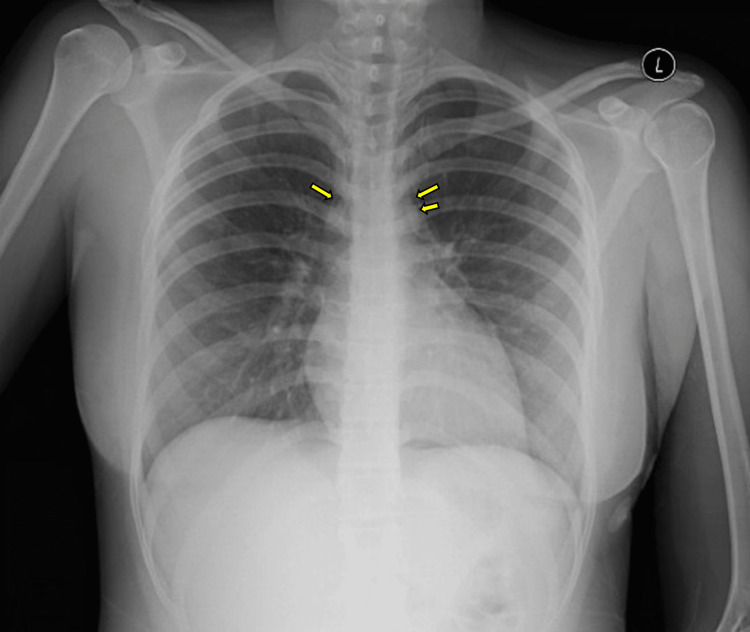
Chest radiograph of a PM Posteroanterior chest radiograph showing probable PM with clear lungs and no pleural effusion or pneumothorax. Arrows denote lucent streaks of extraluminal air in the mediastinum PM: pneumomediastinum

A chest radiograph and CT scan were obtained for diagnostic evaluation. A chest radiograph in the ED showed a probable PM with clear lungs and no pleural effusion or pneumothorax (Figure 1).

**Figure 2:**
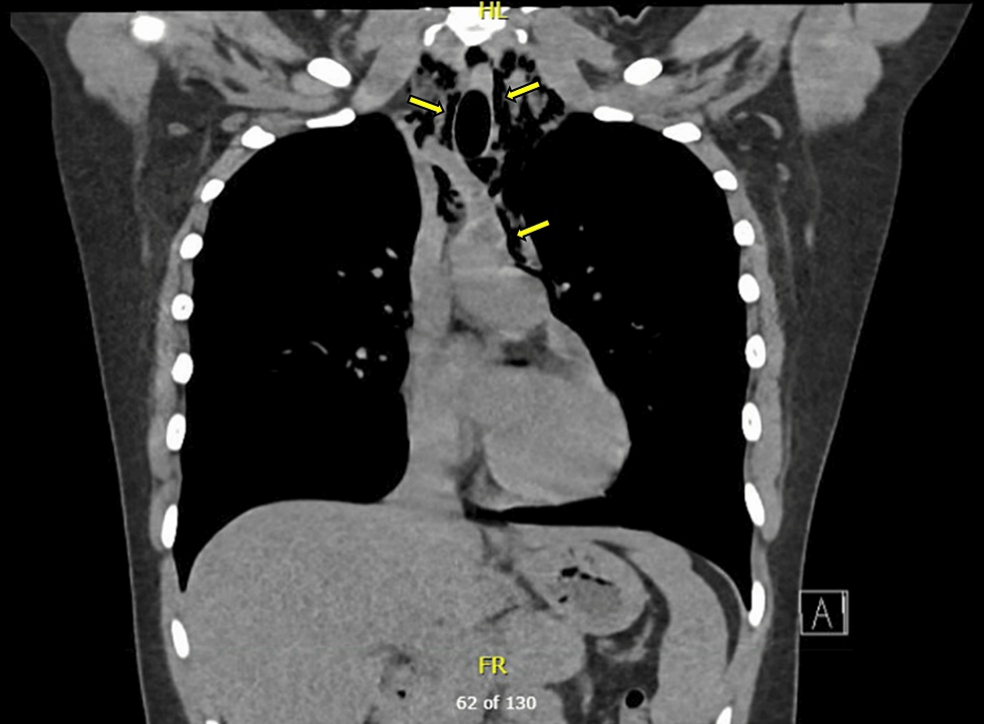
CT chest of a PM with subcutaneous emphysema extending into the neck Coronal CT chest with a mediastinal window showing PM with subcutaneous emphysema extending into the neck. Arrows denote free air accumulation within the mediastinal space, reaching the thoracic outlet and surrounding the paratracheal neck space CT: computed tomography; PM: pneumomediastinum

**Figure 3:**
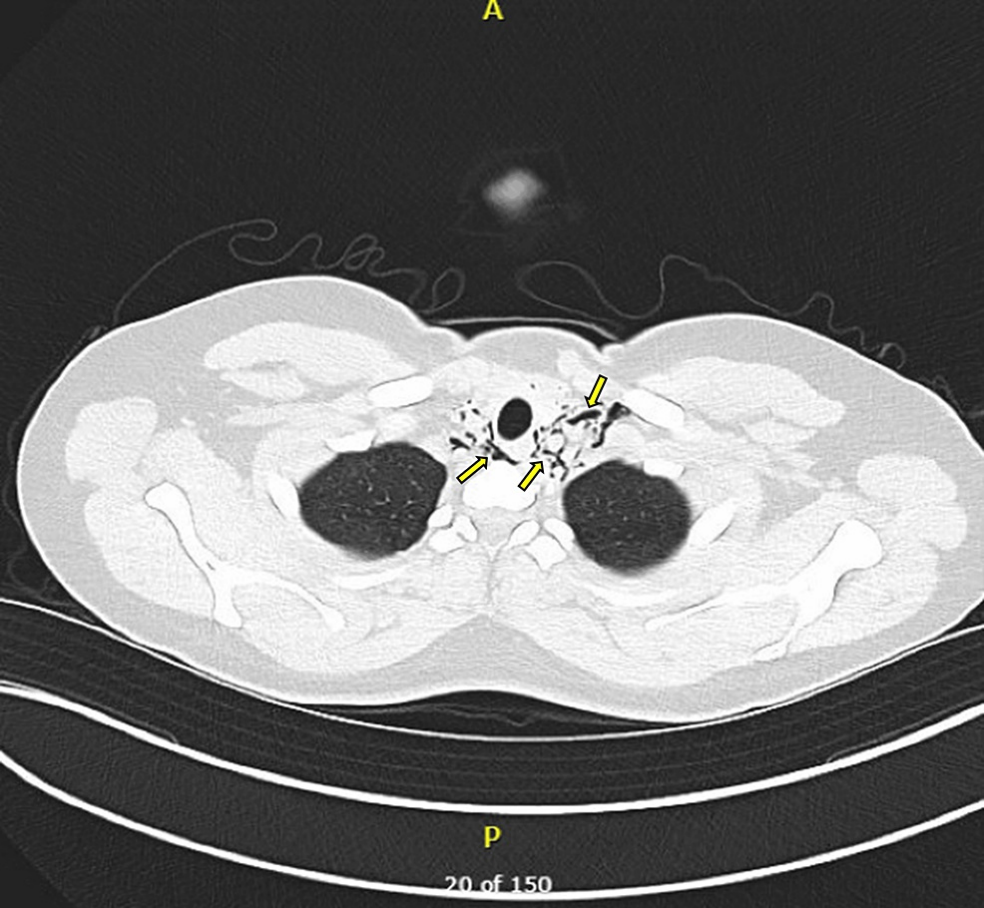
CT neck of subcutaneous emphysema extending into the neck Axial CT neck with soft-tissue window demonstrating subcutaneous emphysema extending into the neck. Arrows denote air pockets surrounding the carotid and paratracheal neck space and subcutaneous tissue CT: computed tomography

A CT chest and a CT neck were obtained to further assess the extent of the PM, which confirmed PM with subcutaneous emphysema extending into the neck (Figures 2, 3).

She received treatment with an albuterol/ipratropium nebulizer, and her dyspnea improved slightly. However, she continued to have persistent dyspnea and tachycardia and was then admitted for further evaluation and close monitoring of her PM.

Her hospital course included conservative management with intravenous methylprednisolone, albuterol/ipratropium inhalations, oxygen, ondansetron, promethazine, benzonatate, and pain management, in addition to oseltamivir. Considering the patient’s acute emesis and pressure-like chest pain, a Gastrografin esophagogram study was obtained and found to be negative for esophageal tear. Daily chest radiographs showed persistent, though improved, small PM. Her fever and symptoms had resolved, and her respiratory status improved. She was discharged on the second day with close follow-up with a pulmonary clinic.

## Discussion

We report a case of SPM in the setting of influenza A infection and e-cigarettes. Only four case reports presented SPM with influenza A infection in adults 33-60 years old with no significant medical history or other documented risk factors [11]. Three cases reported e-cigarette or vaping-induced SPM, while only one case presented SPM in the setting of e-cigarette use and untreated influenza B infection [6-9]. Our case report represents the only case of SPM in the setting of influenza A infection and e-cigarette use. However, the interplay between influenza A infection and e-cigarettes in precipitating SPM is unknown.

Without trauma or underlying airway disease, influenza A infection is likely an etiology in our patient’s SPM. Her associated cough and vomiting episodes may have precipitated the high pressures needed to rupture alveoli and introduce air in the perivascular sheaths. Across all cases, management was conservative, including supplemental oxygen and oseltamivir to treat the viral infection. However, despite the lack of information surrounding the habits of e-cigarettes and the temporal nature of use and symptom onset, our patient’s history of e-cigarette use may also be an underappreciated risk factor that may predispose the alveoli to rupture.

The Valsalva maneuver and Müller’s maneuver are common maneuvers used with inhaled substance use such as e-cigarettes, which may have provoked SPM. Historically, repeated Valsalva maneuvers have been known to cause SPM. Therefore, it may be possible that performing repeated Valsalva maneuvers of deep inhalations and holding the smoke in the lungs during e-cigarette use may increase intrathoracic pressure against a closed glottis and result in alveolar rupture [1,2,12]. Alternatively, Müller's maneuver may also be utilized. Repeated deep inspirations through a high resistance, narrow tube exhibit Müller's maneuver. Consequently, a transmural gradient from the deeply negative intrathoracic pressure causes overdistention and alveolar rupture [12]. Hazouard et al. reported a patient with SPM after repeated deep inhalations through a narrow, high-resistance tube of a bong apparatus during marijuana inhalation, which resembled Müller's maneuver [12]. It is possible that our patient may have used an e-cigarette system with features resembling a narrow, high-resistance tube while performing repeated Müller's maneuvers. Both Valsalva and Müller's maneuvers may have been cycled during e-cigarette use in our patient and possibly introduced extreme pressures to alveoli and predisposed them to rupture.

The inhaled aerosolized mixture from e-cigarettes has a milieu of effects on the lungs, which may cause possible genetic and cellular changes to the airway and alveolar epithelia, as well as damage from direct toxic injury and inflammation that may affect the lungs at various levels. E-cigarettes combust an “e-liquid” that results in an “e-vapor” (EV), which is then inhaled. The aerosol is mixed with flavoring solvent particles, such as propylene glycol and glycerin, and a psychoactive compound, such as nicotine, THC, or both [13,14]. Studies have identified flavoring aldehydes that mediate the production of reactive oxygen species in monocytes and increase oxidative stress in various lung cells. In alveolar type-I and type-II epithelial cells, EV has been shown to reduce mitochondrial function and, thus, increase oxidative stress, resulting in apoptosis or reduced viability [15]. Moreover, EV increases IL-8 levels, recruiting neutrophils, which further contributes to inflammation and oxidative stress [14,15]. EV has also been shown to decrease the expression of tight junction proteins, causing airway epithelial barrier dysfunction through increased inflammatory signaling. Additionally, the resultant increase in permeability may contribute to increased susceptibility to infection [16]. Flavorings containing diacetyl are known to decrease ciliogenesis by decreasing the expression of cilia-related genes and may cause reduced mucosal clearance, precipitate a cough, or foster bacterial growth [14]. Formaldehyde, a breakdown product of propylene glycol and glycerin, is a mucosal irritant associated with bronchitis and pneumonia. Furthermore, additives in the vape liquid, such as vitamin E, a thickening agent commonly found in THC-containing vape liquid, and certain flavoring aldehydes have been shown to disrupt pulmonary surfactant, increasing alveolar surface tension [14,16].

EV may reduce the overall integrity of the alveoli and airway at the genetic and cellular levels, making the alveoli susceptible to rupture. Furthermore, breathing mechanics associated with e-cigarette use may also predispose alveoli to rupture by introducing extreme pressure gradients. Consequently, our patient with influenza A infection exhibited coughing and vomiting episodes, likely creating an extreme pressure gradient. This led to the rupture of weakened alveoli, potentially predisposed by e-cigarettes, ultimately allowing air to enter the interstitium.

A limitation of our case report was the lack of documentation on the habits of e-cigarette use and the temporality between e-cigarette use and symptom onset, as well as other details surrounding e-cigarettes. Therefore, we highlight the need for a detailed history of e-cigarettes when determining the etiology of SPM. A detailed history of e-cigarette use may include the habits of e-cigarette use, the temporality of e-cigarette use and symptom onset, the type of e-cigarette system used with detailed features, breathing patterns employed, and quantity of breaths, flavorings, and mixtures involved, particularly THC, and the concurrent use of illicit substances. In addition to conservative management and addressing the underlying causes, patients should be counseled on quitting e-cigarettes and illicit substances if considering substance use as a possible factor.

We report a 20-year-old previously healthy female with a history of influenza A infection in 2021. She presented to the emergency department (ED) after two days of gradual onset of shortness of breath, cough, wheezing, and sore throat associated with fever, chills, fatigue, body aches, pressure-like chest pain, nausea, and multiple episodes of non-bloody, nonbilious emesis.

## Conclusions

Our case report presents SPM in a previously healthy 20-year-old female with untreated influenza A infection and a history of e-cigarette use. The patient’s cough and vomiting episodes associated with influenza A infection may have created an extreme pressure gradient that ruptured the weakened alveoli and introduced air into the interstitium. The weakened alveoli may have resulted from the reduced alveolar integrity caused by the EV and breathing patterns associated with e-cigarettes. With a rise in e-cigarette use among teenagers and young adults, it is important to actively query a detailed history of e-cigarette use when determining the etiology of SPM. A detailed history of e-cigarette use may include the habits of e-cigarette use, temporality of e-cigarettes and symptom onset, the type of e-cigarette system used with detailed features, breathing patterns employed and quantity of breaths, flavorings and mixtures involved, particularly THC, and the concurrent use of illicit substances. Management for SPM is conservative and should include addressing underlying etiologies with special attention to cessation and education of e-cigarettes and illicit substances.
